# Remote Sensing-Based Outdoor Thermal Comfort Assessment in Local Climate Zones in the Rural–Urban Continuum of eThekwini Municipality, South Africa

**DOI:** 10.3390/rs15235461

**Published:** 2023-11-22

**Authors:** Terence Darlington Mushore, John Odindi, Rob Slotow, Onisimo Mutanga

**Affiliations:** 1 Discipline of Geography, School of Agricultural, Earth and Environmental Sciences, University of KwaZulu-Natal, P/Bag X01, Scottsville, Pietermaritzburg 3209, South Africa; 2 Department of Space Science and Applied Physics, Faculty of Science, University of Zimbabwe, MP167, Mt Pleasant, Harare 00263, Zimbabwe; 3 Oppenheimer Fellow in Functional Biodiversity, Centre for Functional Biodiversity, School of Life Science, University of Kwazulu-Natal, Pietermaritzburg 3209, South Africa; 4 Department of Genetics, School of Genetics, Evolution & Environment, University College, London WC1E 6BT, UK

**Keywords:** urban climate, heat stress, thermal comfort, remote sensing, WUDAPT, temperature variability

## Abstract

Due to the need to continuously monitor and understand the thermal environment and its socioeconomic implications, this study used remotely sensed data to analyze thermal comfort variation in LCZs, including along the rural to urban gradient of the eThekwini Municipality in KwaZulu-Natal province of South Africa. LCZs were mapped using multi-temporal and multi-spectral Landsat 8 and Landsat 9 data using the approach by World Urban Database and Access Portal Tools (WUDAPT), while thermal data were used to retrieve land surface temperatures (LSTs). Data for training classification of LCZs and accuracy assessment were digitized from GoogleEarth guided by knowledge gained and data collected during a field survey in March 2022 as well as pre-existing maps. LCZs were mapped using the random forest classifier in SAGA GIS software while a single channel algorithm based on band 10 was used to compute LST for different scenes. The LSTs were adjusted and further used to derive thermal comfort based on the Universal Thermal Comfort Index (UTCI) categories as an indicator for outdoor thermal comfort on the extremely low- and extremely high-temperature periods in the cool and hot seasons, respectively. LCZs were mapped with high accuracy (overall accuracy of 90.1% and kappa of 0.88) while inter-class separability was high (>1.5) for all LCZ pairs. Built-up LCZs dominate the eastern parts of the municipality, signifying the influence of the sea on development within the area. Average LST was coolest in the dense forest, open low-rise and water LCZs in the cool and hot seasons, respectively. The compact high-rise LCZ was the warmest in both the hot (36 °C) and the cool (23 °C) seasons. The sea sands were among coolest regions in both seasons due to their high water content, attributed to their high water table and close proximity to the ocean. There was no thermal stress during the cool season, while most areas recorded moderate to strong heat stress in the hot season. Some areas in the densely built-up LCZs recorded very strong heat stress in the hot season. The findings suggest that policies and strategies should enhance heat mitigation capacities in strong-heat-stress areas during the hot season. Municipal authorities and citizens must work together to build strategies to minimize temperature extremes and associated socioeconomic pressures. Urban development policies, plans and strategies should consider implications on the thermal environment as well as the value of conservation of LCZs with high-heat mitigation value such as dense forests and expansion of built-up LCZs with low-heat absorption levels such as open low-rise. The study was based mainly on remotely sensed temperatures with some ground data used to validate results, which may limit the assessment. Overall, the study provides insights towards achievement of global sustainable and climate-smart development targets.

## 1 Introduction

Temperature extremes (both high and low) affect energy usage as demand for space warming or cooling increases, especially in areas occupied by human residences and socioeconomic activities [1–4]. Studies have also revealed that extreme temperature can affect the concentration of teenagers in schools [5] and reduce performance in workplaces [6]. Warming commonly increases costs and demand for water for household and non-household needs [7]. In countries with drinking water shortages, heat extremes increase the risk of water-borne diseases as demand rises beyond supply [8]. Furthermore, warming is associated with heat-related diseases, stress and even death [9,10]. For instance, high temperatures can cause or worsen cardiovascular diseases increasing levels of health burdens amidst other ailments [11]. Based on studies in the United States, heat extremes are associated with mental health impacts that include increased irritability and depression and an increase in suicides [12,13]. Hence, due to the projected rise in temperatures, there is a need to continue monitoring and understanding temperature patterns and their associated impacts [8].

Remote sensing provides capabilities to monitor and quantify temperature changes at various spatial and temporal scales. For instance, missions such as Landsat offer moderate resolutions which provide detailed analysis dating back to 1972 [14]. Moderate resolution datasets enable linking thermal–spatial patterns to other surface properties such as land use/land cover (LULC) at corresponding periods [15]. Previous studies which related land-surface characteristics to LST the mostly used generic land-use–land-cover classes, which are subjective, site specific and not categorized based on climate. On the other hand, remotely sensed-based local climate zone (LCZ) maps partition an area into globally standardized themes that are directly related to climate to remove the subjectivity and area specificity associated with general LULC schemes [16]. LCZs are regions of homogeneous surface-air temperature covering hundreds to ten thousands (10^4^) of square meters in the horizontal scale [17,18]. Hence, the use of LCZs allows global comparison and in-depth understanding of the effect of LULC patterns on the thermal environment [16,18]. Additionally, the use of LCZs is an important contribution to the World Urban Database and Access Portal Tool (WUDAPT) [19]—with potential to contribute to sustainable-development planning.

Studies have related LCZ to urban temperatures and heat islands [20–24]. An LCZ scheme is also useful in developing globally standardized approaches for quantifying surface urban heat island (UHI) intensities compared to the subjective urban–rural temperature difference method [20,22,25]. Whereas LCZs are effective for mapping and characterizing the thermal environment, they have mainly been used for characterizing the thermal environment in urban areas while ignoring their potential to influence thermal variabilities in the rural–urban continuum. The rural–urban continuum allows comparison of thermal characteristics of undisturbed and natural surfaces with more urbanized areas. The gradient also contains variations in the extent of transformation between the purely rural and the purely urbanized areas. Rural areas may also provide a more natural landscape as a reference point for the local transformed areas, and may provide a local heat sink and mitigate urban heat build-up [26]. The thermal variability analysis continuum helps to identify spatial variations of heat-stress levels as well as establish potential mitigation mechanisms and, thus, options based on the association between LCZ and respective thermal properties.

The eThekwini Municipality is characterized by a wide variation in surface characteristics including different vegetation types, built environments, and exposed soil proportion along the rural–urban gradient that is worthy of in-depth analysis. To date, there is a lack of LCZ maps covering the rural–urban continuum. In South Africa, a sole study by Sithole and Odindi [27] determined the correlation between land surface properties and thermal variability in the rural–urban continuum in Pietermaritzburg city. However, the study used generic LULCs and not LCZs. Another study within the eThekwini area by Odindi et al. [28] focused on the heat mitigation function of urban green spaces and compared thermal behavior of the area with other coastal cities in the southeast coast of South Africa [29]. However, the eThekwini Municipality in South Africa contains a rural–urban continuum with a heterogeneity of LCZs and thermal environments that require characterization. Also, no study within the study area has explored the influence of LST variations on outdoor thermal comfort in the rural–urban gradient. This is important to express the extent to which thermal comfort can be influenced by the extent of ruralness or urbanness.

To date, attention has largely focused on indoor thermal comfort, as people, especially in the developed world, where most of these studies were undertaken, spend much of their time indoors [30]. Additionally, some outdoor climates previously regarded as beyond adjustment can now be controlled [30]. Increasingly, attention is now placed on outdoor thermal comfort as people carry out essential services outdoors (e.g., road repair, garbage disposal and policing), outdoor industrial or business activities (e.g., road construction and market places) as well as recreational activities such as sports [8]. While most space-borne remote sensing-based thermal analyses provide instantaneous conditions, they are limited by temporal resolution in depicting conditions at the desired times of the day. For instance, the overpass time may not coincide with the lowest or highest temperature of the day, thus rendering some data incapable or revealing thermal comfort patterns at moments of temperature extremes. Extremes are important for planning as they represent the worst-case scenario for preparedness. There is paucity of studies that adjust temperature data to represent thermal comfort at times when there is no satellite overpass, to the best of our knowledge. In most cases, the overpass time may not coincide with the times of lowest or highest temperature extremes implying the need to develop methods to assess thermal comfort under such extremes. This also helps to quantify the differences in the thermal behaviors of LCZs under very low- and high-temperature conditions.

In view of the above and the need to ensure the thermal comfort of residents during both cold- and hot-season extreme temperatures, using remotely sensed moderate resolution data from Landsat 8 and Landsat 9 satellite missions, the objectives of this study were to (i) map local climate zones and land surface temperature variability in the rural–urban continuum of eThekwini Municipality in South Africa; (ii) to develop a technique for estimating thermal properties at the peak temperature time when this does not coincide with satellite overpass; (iii) assess outdoor human thermal comfort in relation to LCZs using time-adjusted temperatures in the rural–urban continuum of eThekwini Municipality. In this study, LCZ and LSTs will be mapped and used to explain intra- and inter-LCZ variations in thermal comfort. The study will also explore changes in the thermal behaviors and comfort conditions along the rural–urban continuum using LST as a proxy for outdoor thermal comfort in eThekwini municipality.

## 2 Materials and Methods

### 2.1 Study Area, the eThekwini Municipality

**Figure 1:**
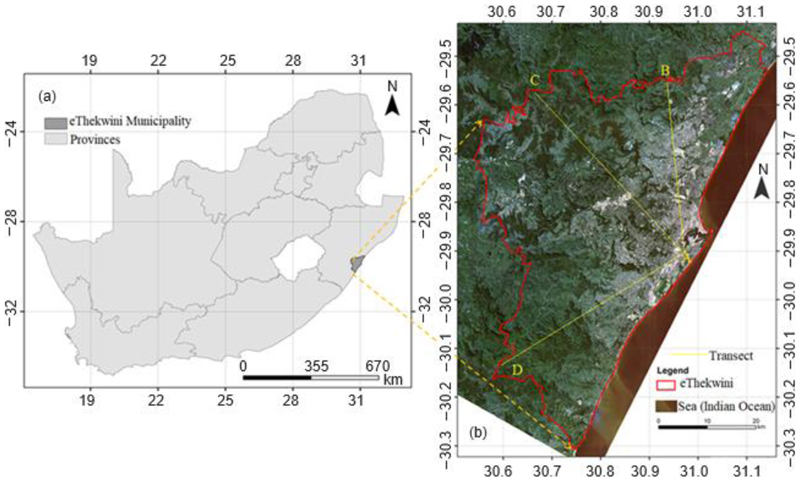
Map of South Africa showing eThekwini Municipality (**a**) and Landsat image showing variation in surface properties within the municipality and its surroundings (**b**). (**b**) Shows the general variation in vegetation cover with dense vegetation shown in deep green. The grey shades represent built-up areas with brightness increasing with increase in built-up density. Generally, there is an eastward increase in the built-up proportion. (**b**) Also shows, in yellow, Transect 1 (B to A), Transect 2 (C to A) and Transect 3 (D to A), along which changes in temperature from rural to urban areas will be demonstrated.

Assessment of the interaction between LCZs and the thermal environment was performed in the eThekwini Municipality (Figure 1a) in KwaZulu-Natal Province, South Africa. The green areas (Figure 1b) are occupied by very sparsely built-up local climate zones such as farmlands and rural homesteads as well as forests of various densities. The area experiences a humid subtropical climate (Cwa) with a cool and dry winter (May to October) as well as a hot and wet summer (November to April) based on the Koppen classification [28]. Annual precipitation ranges from 500 to 2000 mm with a typical average of 1000 mm. In summer, daily temperatures range from 20 to 35 *°*C and 12 to 25 *°*C in winter. It is this wide variability between and within the cool and hot seasons which necessitates analysis of thermal comfort to identify hot and/or cold spots in both seasons. The municipality occupies an area of 2212 km^2^ with a density of 1513 people/km^2^, with Durban city having the highest population density, accommodating 35% of the population of the municipality [28].

### 2.2 Remotely Sensed Data

Landsat 8 and Landsat 9 level 1 data were downloaded from the United States Geological Survey (USGS) earth explorer website (https://earthexplorer.usgs.gov/ accessed on 16 February 2023). For Landsat 8 and Landsat 9, reflective data are acquired at a spatial resolution of 30 m (except the 15 m resolution panchromatic bands) while thermal data were acquired at 100 m but downloaded at a resampled resolution of 30 m. Landsat 8 and Landsat 9 have a temporal resolution of 16 days, which limits their use in expressing very rapidly changing spatial phenomena such as hourly patterns. The actual imageries and dates of acquisition are shown in Table 1. The study area could not be accommodated in a single Landsat scene. Therefore, scenes with path/row 168/80 and 168/81 were mosaicked to provide a complete analysis for the municipality for the corresponding dates (Table 1). Atmospheric correction was performed using the dark subtraction technique for all dates [31,32]. The corrected images constituted reflectance layers for respective wavelength ranges which were then compiled into a single multi-layer file that was used for mapping of LCZs for the municipality. Since some LCZs, such as those based on vegetation, change properties in seasonal cycles, use of multi-temporal data was necessary to capture the characteristics of seasonality to aid the classification procedure. Thermal infrared data were left out in LCZ mapping as they were reserved for retrieval of LST for the different dates and seasons.

### 2.3 Local Climate Zones Samples for Classification and Accuracy Assessment

Table 2 provides the depictions of LCZs identified in the study area guided by standard description of LCZ in the literature [33]. Some of the classes were similar and were thus merged into the same LCZ category. For example, due to the very low density of buildings in rural areas, it is difficult to separate the sparsely built areas from the low plants LCZs. In other cases, there was co-existence of different LCZs within a 100 m by 100 m area, which is the minimum area of a land surface temperature pixel from Landsat 8 and Landsat 9 thermal data. This was mainly observed in the business district where tall buildings could be found in the same area with mid-rise and low-rise buildings in a highly impervious area. In such cases, the area was assigned to the category with the most pixels. Pre-existing LULC maps, such as the 10 m resolution global map from the European Space Agency, as well as knowledge of the study area gained during a field survey in March 2022, guided digitization of training polygons in GoogleEarth. For LCZs with a large spatial extent, a minimum of 20 polygons were digitized from across the study area to capture intra- and inter-class variability and increase discriminability. For each class, samples were collected using the WUDAPT procedure [34] at locations randomly selected around the study area in order to capture as much of within-class variability as possible. Another set of polygons was digitized and set aside as independent data for post-classification accuracy assessment.

### 2.4 Local Climate Zone Mapping and Accuracy Assessment

Multi-temporal remotely sensed data from Landsat 8 and 9 reflective bands were processed into a single multi-layer file using the layer stacking tool in ENVI software. This was carried out to create spectral signatures sufficient to group pixels with similar properties into the same LCZ. Furthermore, multi-temporal data also considered seasonal variation especially in vegetation- and water-based LCZs. Supervised image classification was performed using the random forest (RF) classifier to assign LCZ labels to pixels guided by training samples. The RF classifier makes use of random bootstrapped training data to build multiple trees in an ensemble learning technique [35,36]. The RF classifier was chosen as it achieves better results than other classifiers such as support vector machines (SVMs) when multi-dimensional data (such as multi-temporal and multispectral data) are used [37]. Unlike SVM, RF is also superior at handling unbalanced training and missing data [38]. The RF classifier has few parameter settings and is capable of computing variable importance to enhance classification accuracy [39]. A LCZ map for eThekwini was produced and validated against an independent set of LCZ samples digitized from GoogleEarth. Accuracy assessment was then performed by cross tabulation (confusion matrix), which compared class labels assigned to pixels with their ground truth value from high resolution GoogleEarth retrievals [32]. An independent set of ground truth data regions of interest (digitized polygons for each LZC category) was used for accuracy assessment. Similar to Stefanov et al. [40], Ahmed and Ahmed [41] and Danylo et al. [42], the matrix was used to derive accuracy indicators which included producer accuracy, user accuracy, overall accuracy as well as the kappa statistic.

### 2.5 Mapping of Land Surface Temperature Using Thermal Infrared Data

Land surface temperatures for the cool and hot seasons were derived from Landsat 8 and Landsat 9 thermal data. For each date, a single channel algorithm [43] was used to compute temperature from Band 10 due to inaccuracy associated with Band 11. Band 11 is highly sensitive to water vapor and has large calibration uncertainties, hence the use of Band 10 [44]. Firstly, digital numbers (DN) were used to compute radiances using Equation (1) [45–47].

(1) Radiance=Gain×DN+Offset

Gain was 0.0003342 and 0.00034 for Landsat 8 and Landsat 9, respectively, while they had the same value for offset (0.1) obtained from metadata files downloaded with the data. The radiances were used to compute brightness temperatures (*T*_*B*_) using Planck’s algorithm for Landsat in Equation (2) [46].

(2) $$T_{B}=\frac{K_{2}}{\text{ln}(\frac{K_{1}}{\;Radiance\;}+1)}-273.16$$

Brightness temperature is obtained assuming that all surfaces are blackbodies with equal emissivity, which is not the case in reality. A further step of emissivity correction is carried out to eliminate this assumption by assigning emissivity values to pixels depending on the type of surface occupying them. *K*_1_ and *K*_2_ are constants which take values of 774.8853 and 1321.0789 when using Band 10 of Landsat 8, and 799.0284 and 1329.0284 when using Band 10 of Landsat 9. The coefficients were taken from metadata files which accompany the respective images. The vegetation proportion (PV) obtained using Equation (3) was used as an input in Equation (4) to obtain an emissivity layer [48].

(3) $$PV={\left[\frac{NDVI-NDVI_{soil\;}}{NDVI_{vegetation\;}-NDVI_{soil\;}}\right]}^{2}$$

(4) Emissivity=0.004×PV+0.986

NDVI, NDVI_min_ and NDVI_max_ are the value for each pixel in the normalized difference vegetation index image, the value for a soil pixel and the value for a vegetation pixel, respectively [48]. NDVI_min_ and NDVI_max_ correspond to values for pure soil and vegetation pixels taking values of 0.5 and 0.2, respectively, based on Subrino et al. [49]. The emissivity correction is performed to obtain LST in degrees Celcius using Equation (5) [43,45,49]

(5) $$LST=\frac{T_{B}}{\left(1+\frac{\lambda T_{b}}{\alpha }\right)\;\ln(Emmissivity)}$$

*λ* is the central wavelength which is 10.9 µm for the thermal data (Band 10) of Landsat 8 and Landsat 9 while α takes a value of 1.438 *×* 10^*−*2^ mK [50]. The multi-date LST maps were used to display variations with time in the different months of the two seasons. Since the imageries represented the data available on cloud-free days in both seasons in 2022, multi-date data helped to identify the warmest and the coolest scenes in the hot and cool seasons, respectively. This aided analysis and understanding of thermal comfort under hot and cold temperature extremes.

### 2.6 Adjustment of LSTs to Represent Thermal Conditions at the Minimum and Maximum Daily Temperature

The overpass of Landsat 8 and Landsat 9 in eThekwini is at 0950 hours while the missions have 16-day cycles. This timing misses the hours of very low temperature overnight and very high temperature during the day. These hours represent extremes of thermal comfort, especially the hottest hours of the day in the hot season and the coolest hours of the day in the cool season. Hence, there was a need to adjust the LSTs in order to depict the spatial patterns of these extremes. Since the data made available by the South African Weather Services was at daily resolution and not sufficient for detailed temporal analysis, we used temperature data for Ushaka weather station at 5 min resolution, from Ethekwini Datafeeds website (https://data.ethekwinifews.durban/instrument/stations, accessed on 30 May 2023) maintained by eThekwini Municipality. Ushaka weather station was selected as it was the only station within eThekwini with high-temporal-resolution temperature data for the analyzed dates. Ushaka station is also located in a hot area making it representative in the analysis of high temperature extremes. For dates corresponding to Landsat scenes in Table 1, maximum temperature (T_max_) and temperature at 0950 hours (T_0950hours_) were extracted. The difference between maximum temperature and temperature at 0950 hours at Ushaka weather station was used to estimate change in LST from 0950 hours to its peak. The adjustment was applied to obtain maximum LST for a day_*i*_ corresponding to each scene LST (i) using Equation (6) developed in this study for that purpose.

(6) $$LST_{max}(i)=LST(i)+\left[T_{max}(i)-T_{0950hours}(i)\right]$$

LST_max_ was the estimated peak of the land surface temperature for the day. LST_max_ was needed in order to map temperature and comfort patterns at extreme temperatures instead of the overpass time, which was at mid-morning. The rate of change in temperature at Ushaka was assumed to be the same as that of the entire eThekwini Municipality. Ideally, either moderate spatial resolution temperature data or weather stations in the different LCZs are required to accurately derive changes in LST per LCZ category. However, moderate resolution remotely sensed data has low temporal resolution.

### 2.7 Linking LCZ to LST and Thermal Comfort Variation in eThekwini

Each LCZ category was converted to points in order to extract LST values using the ‘Extract values to points’ tool in ArcGIS 10.2 software. The extracted values were used to generate box plots to show the variations of LSTs between and within LCZs. One-way analysis of variance (ANOVA) was used to ascertain if the differences in mean LSTs for the LCZs were significant. Visual inspection of maps was also used to determine the variations of LST with LCZ qualitatively. Thermal indices used for outdoor comfort analysis include the predicted mean vote (PMV), predicted equivalent temperature (PET), outdoor standard equivalent temperature (OUT-SET) and the universal thermal climate index (UTCI) [11,51,52]. The indices are based on data assessments that were performed when individuals and groups were subjected to various levels of environmental as well as metabolic stress factors [53]. While PET and PMV are the most widely used, indices such as the UTCI are also becoming popular. The UTCI has also been found to be strongly correlated with the superior PMV, OUT-SET and PET, and is thus of reasonable applicability [54]. The UTCI was developed to create standard conditions suitable in all climates and independent of human metabolism and performs in various climate conditions better than other indices [11]. The UTCI can provide a direct measurement of the implications of different LST classes on urban health scenarios [25]. Contrary to thermal comfort assessment methods such as PMV, PET and OUT-SET, simple algorithms for the UTCI have been developed and tested for remotely sensed data use [25]. Xu et al. [55], for instance, used the UTCI to establish outdoor thermal comfort variability in China. The approach involved complex modeling of relative temperature, pressure, relative humidity and wind speed and further derivation of UTCI (Equation (7)).

(7) $$UTCI=T_{air\;}+f(Wind\;speed,\;pressure,\;Relative\;humidity,\;Relative\;temperature)$$

where *T*_*air*_ is the air temperature.

In another approach, Duta et al. [25] reclassified LST values according to UTCI categories to assess outdoor thermal comfort in New Delhi, India. The approach by Dutta et al. [25] was parsimonious due to the requirement of LST data being the only input while managing to map spatial variations in thermal comfort.

Duta et al. [25] reclassified LST into 10 thermal comfort categories of the UTCI, namely extreme heat stress (>46 *°*C), very strong heat stress (38 to 46 *°*C), strong heat stress (32 to 38 *°*C), moderate heat stress (26 to 32 *°*C), no thermal stress (9 to 26 *°*C), slight cold stress (0 to 9 *°*C), moderate cold stress (*−*13 to 0 *°*C), strong cold stress (*−*27 to *−*13 *°*C), very strong cold stress (*−*40 to *−*27 *°*C) and extreme cold stress (less than *−*40 *°*C). Similarly, the UTCI categories can be used to map thermal comfort in the LCZs of the rural–urban continuum of eThekwini municipality given that it can be adopted in a range of climates. LST_max_ for the warmest scenes in the cool and hot seasons obtained from the data in Table 1 was used to assess daytime thermal comfort under the extreme temperatures in eThekwini.

### 2.8 Potential Sources of Uncertainty in the Methodology

The study used temperature variations at Ushaka to build an adjustment factor for the entire municipality. Temperature patterns should, however, also vary with location requiring point specific adjustment. This could not be undertaken due to unavailability and inaccessibility of high-resolution in situ temperature data for different locations in the area. The other accessible data had low temporal resolution and no values at times coinciding with the overpass of Landsat. While Landsat data has reasonable spatial resolution, its temporal resolution limited actual analysis of extremes. The UTCI involves complex steps of computation. In this study, LST values were reclassified based on UTCI ranges while actual computation of the UTCI would require other details such as pressure, relative humidity and wind speed. There were no spatial data of these additional parameters, which limited the analysis. There was also no mechanism to assess the extent to which LST values differ from the UTCI.

## 3 Results

### 3.1 The Local Climate Zones Map of eThekwini

**Figure 2:**
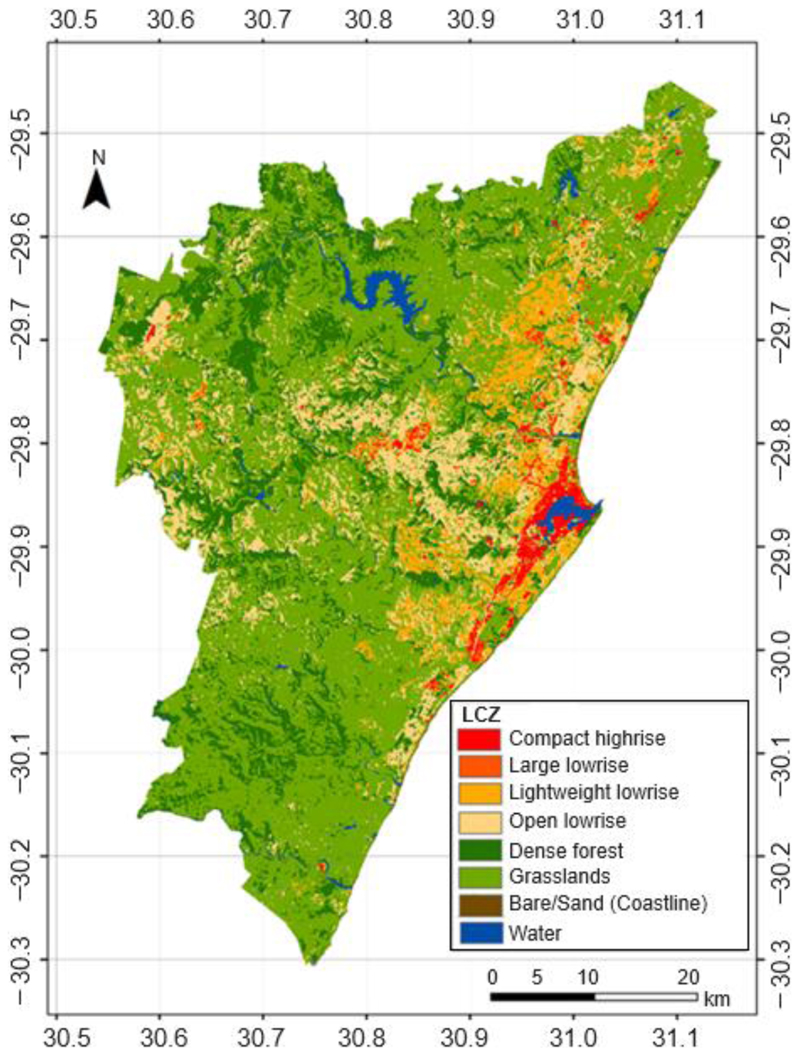
Local climate zones map of the rural–urban gradient of eThekwini Municipality.

Based on visual inspection, the vegetation-based LCZs dominate northwestern and southern areas of eThekwini municipality. The built-up LCZs cluster towards the eastern margins of the municipality and along the coast (Figure 2). Small clusters of built-up LCZs, especially the open low-rise, are also scattered within the study area. In some places, densely built-up LCZs such as compact high-rise and large low-rise exist within low-density buildings.

The beach sands accounted for a very small proportion while the low plants LCZ occupied the bulk of the municipality (Table 3). The water LCZ had more coverage than the sand and large low-rise LCZs. The open low-rise LCZ occupied the largest proportion of the municipality compared to the other built-up LCZs. Densely built-up LCZs (compact low-rise, large low-rise and lightweight low-rise) had lower coverage than open low-rise LCZs.

An overall accuracy (OA) of 90.1% and kappa of 0.88 (Table 4) showed that the LCZ map of eThekwini municipality was mapped with reasonably high accuracy. The producer accuracies per class ranged between 64 and 99%, while user accuracies ranged between 83 and 99.4%. Producer accuracy was lowest in large low-rise LCZs (64.9%) and highest in the water LCZ (98.8%). User accuracy was lowest in lightweight low-rise (83.3%) and highest in water (99.4%) LCZs. The water LCZ was thus mapped with the highest accuracy compared with the other LCZs, as indicated by user and producer accuracies above 98%. Producer accuracies were lowest (<75%) in large low-rise and compact high-rise.

Based on the transformed divergence separability index (TDSI) which took values between 1.5 and 2 (Table 5), the LCZs were highly discriminable using the provided regions of interest and Landsat data. Of the built-up LCZs, lightweight low-rise and compact high-rise were the most difficult to separate, although the TDSI value (1.64) was still very high, ensuring the discriminability of the two categories. For most of the LCZ pairs compared, the TDSI was 2, indicating the ease of separation of the two classes.

### 3.2 Temporal Variations of LST in the Hot Wet and Cool Dry Seasons in eThekwini

LST averages across the scene ranged between 17.7 *°*C and 28.9 *°*C for the considered scenes obtained in different months covering the cool and hot seasons. In the cool season, the lowest average LST was 17.7 *°*C, recorded from a scene obtained in August, while the highest was 19.0 *°*C, recorded in July. In the hot season, the average LSTs ranged between 26.2 and 28.9 *°*C with the highest recorded from a scene in February and the lowest in March. Two measurements recorded in March were within 0.5 *°*C of each other. An average temperature difference of at least 7 *°*C was measured between the scenes of the cool and hot seasons.

### 3.3 Hourly Variations in Temperature and Time-Adjusted Land Surface Temperatures

On average, the maximum temperature was about 3.3 *°*C warmer than the temperature at 0950 h (Table 6). Table 6 shows the adjustments applied to LST imageries to obtain maximum temperatures (extremes) for the days corresponding to the overpasses. The adjustment factors were not uniform for different days.

### 3.4 Spatial Variations of LST in the Cool Dry and Hot Wet Seasons

**Figure 3:**
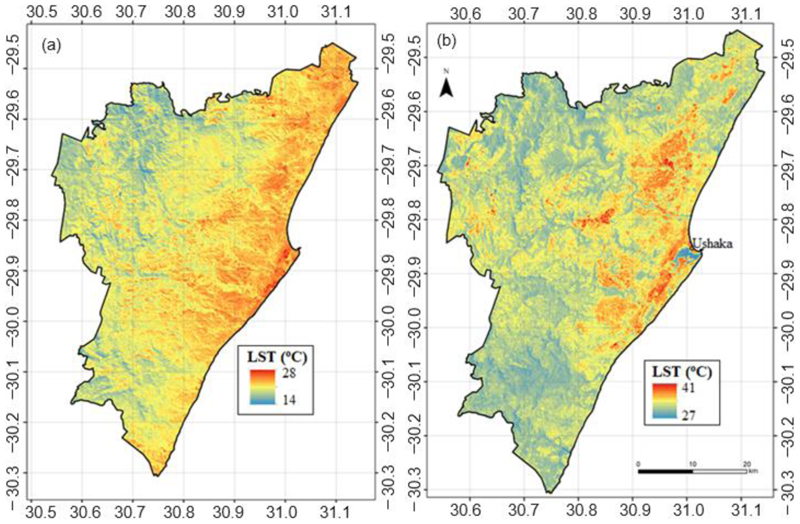
Comparison of LSTs between the cool dry (a) and hot wet (b) seasons under extreme warm temperature conditions. The location of the Ushaka weather station is indicated on (b).

Derived temperature maps corresponding to the daytime peak temperature for warmest days in the cool as well as the hot season are presented in Figures 3a and 3b, respectively. There was a general eastward increase in temperature for both days in the two seasons. The warmest scene among the cool-season temperatures showed limited spatial variations as areas of the same temperature regimes covered wide areas with a marked east and west segmentation. For a hot day in the hot season, temperatures showed wide spatial variations compared to the distribution in the cool season (Figure 3b). Figure 3b also shows that it was generally cooler along the coast than the built-up areas to the immediate west.

### 3.5 Analysis of Variance of LST in the Different LCZs

The calculated F factor was greater than the critical F value for LSTs computed in both the cool and warm seasons. *p* values were also less than 0.05 in all circumstances. Based on this, we rejected the null hypothesis and concluded that mean LSTs of different LCZs were statistically different for the warmest days in the cool and hot seasons.

### 3.6 Thermal Comfort Variations in the Cool and Hot Seasons under Very Cold and Very Hot Temperatures in eThekwini Municipality

**Figure 4:**
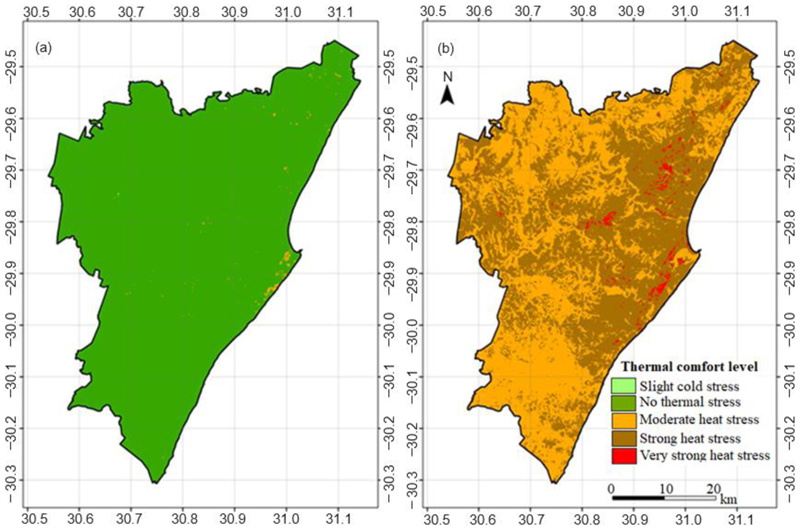
Outdoor thermal comfort on a hot afternoon in the (a) the cool and (b) the hot season based on UTCI levels described in Table 3.

On the warmest hour of the cool season, there was no heat or cold stress in the afternoon, and it was comfortable across the bulk of the municipality. There were only a few locations in the central east, close to the coast, where moderate heat stress was recorded in the cool season (Figure 4a). On the hottest hour of the day corresponding to the hottest scene, moderate to very strong heat stress levels were observed in the study area (Figure 4b). Moderate heat stress was recorded in the northwestern and southwestern parts which corresponded with sparsely (mainly rural) and non-built-up LCZs. Northern and northwestern areas included Inanda, Amalanga, Mlahlanga, Cibane, Imbozamo, Mgangeni and Indanda. Moderate heat stress areas in the south and southwest included Nugwane, Umzinto, Isimahla, Ezimwini, Ogagwini, Mbongolwane and Echobeni. Strong and very strong heat stress categories were recorded mainly in the eastern region. The places here included New Germany, Pinetwown, KwaMashu, Magabeni, Zwelibomvu, Golokodo-Ensimbini, Hammarsdale, Bayhead, Braodway, Siyanda, Glenwood, Prospection Industrial and Durban Central Business District.

### 3.7 Temperature and Thermal Comfort Variations along the Rural–Urban Gradient in eThekwini Municipality

**Figure 5:**
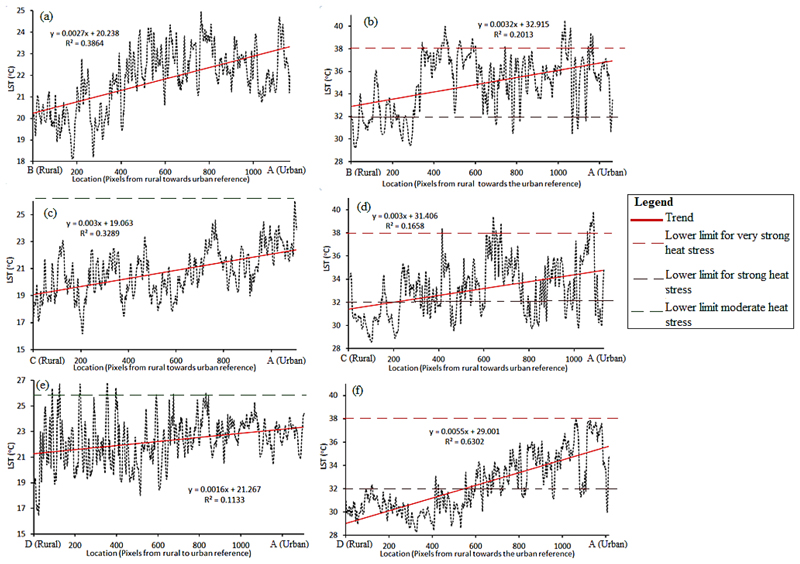
(a–f): Variations in LST along the rural–urban gradient of eThekwini Municipality. Locations A (urban end point) and B, C, D (rural starting points), as well as the line of the three transects are mapped in Figure 1.

In the cool season, the rural and urban temperature difference ranged between 2 and 4 *°*C (Figure 5a,c,e). The difference ranged between 4 and 6 *°*C for a day in the hot season (Figure 5b,d,f). Fluctuations and general rising trends in LST were observed along all transects as we moved from rural to urban areas. Moving from rural areas in the north towards the urban areas (Transect 1) in the central east during a hot day, the LST was low for some distance but rapidly shifted by approximately 4 *°*C higher and fluctuated around 35 *°*C through to the urban representative point (Figure 5b). Along Transect 2, which moved from rural areas in the northwest towards the urban area, the change in temperature on a hot day was more gradual than along Transect 1. A very gradual change in LST was observed along Transect 3 from rural areas in the southwest to the urban area (Figure 5f). Along this transect, the temperature difference was 6 *°*C between the rural and the urban end nodes. Overall, rural–urban LST difference was more marked in the hot than the cool season. In the cool season, thermal comfort conditions did not reach the moderate heat stress level in any pixels along Transect 1 and Transect 2, and only did so for below 1% of a few of the pixels along Transect 3.

On a hot day in the hot season, moderate heat stress areas were observed in the western areas close to the rural reference pixel. There was a tendency towards the strong and very strong heat stress categories as we traversed eastwards towards the urban reference pixel. Along Transect 1, there were some pixels close to the rural reference which recorded strong heat stress while a very strong heat stress level was not recorded around the rural reference along all transects. The category of very strong heat stress became predominant across the urbanized eastern areas.

### 3.8 Intra- and inter-LCZ Variations in Cool and Hot Season Outdoor Thermal Comfort

**Figure 6:**
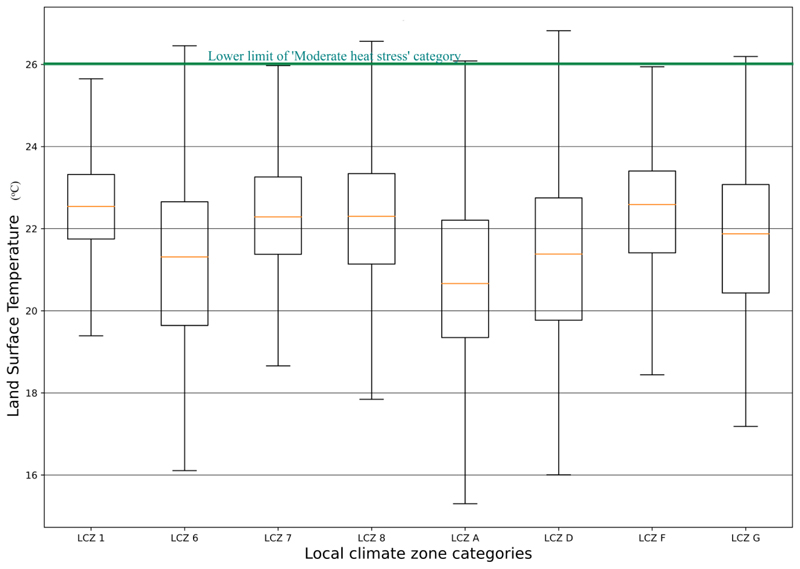
LST variations on the hottest day in the cool season in LCZ1 (compact high-rise), LCZ6 (open low-rise), LCZ7 (lightweight low-rise), LCZ8 (large low-rise), LCZA (dense forest), LCZD (low plants), LCZF (bare soil/sand) and LCZG (water).

Figure 6 shows that on the warmest scene in the cool season, densely built-up local climate zones (compact high-rise, lightweight low-rise and large low-rise) were about 1 *°*C warmer than the sparsely-built-up open low-rise (LCZ 6). The open low-rise LCZ had almost similar LST with low plants (LCZ D) areas while dense forests were the coolest. The water LCZ was warmer than the open low-rise and the dense forest LCZs. In the hot season, lightweight low-rise (LCZ 7) areas were at similar temperature ranges as large low-rise (LCZ 8) areas. In the cool season on a hot day, almost all pixels in the compact high-rise, lightweight low-rise, dense forest, sand and water LCZs experienced no thermal stress. However, there were a few areas (less than 10%) in the open low-rise, large low-rise and low plants areas that experienced moderate heat stress on a hot day in the cool season.

**Figure 7:**
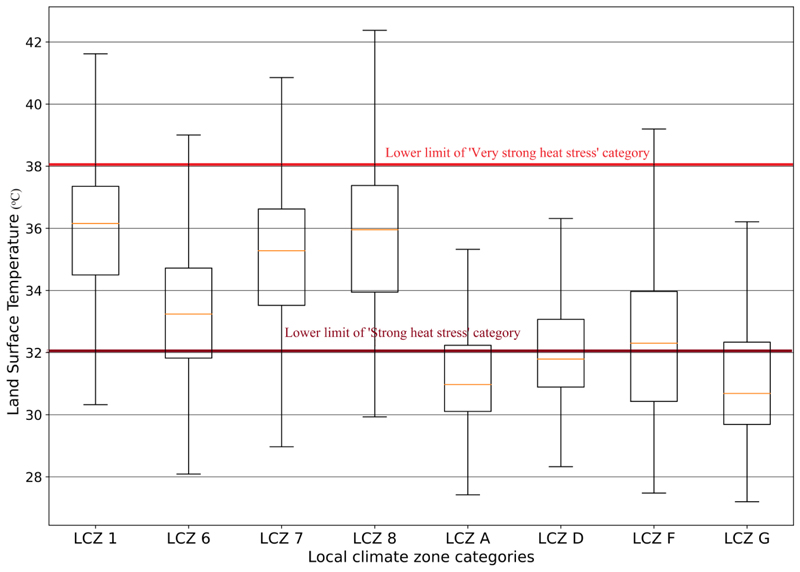
LST variations on an extremely hot day in the hot season in LCZ1 (compact high-rise), LCZ6 (open low-rise), LCZ7 (lightweight low-rise), LCZ8 (large low-rise), LCZA (dense forest), LCZD (low plants), LCZF (bare soil/sand) and LCZG (water).

In a very hot scene in the hot season, compact high-rise was the warmest while water was the coolest LCZ (Figure 7). Open low-rise areas were the coolest of the built-up LCZs. Sand areas were among the coolest LCZs with almost the same temperature as low plants areas, although warmer than dense forest areas. The sand LCZ also had wide variations in LSTs as some of the sands were coastal while others were inland. The sands and bare soils were at least 2 *°*C cooler than the densely built-up and impervious LCZs (compact high-rise, lightweight low-rise and large low-rise). Contrary to observations in the cool season, the large low-rise LCZ was warmer than compact low-rise LCZs. Vegetation- and water-based LCZs were at least 2 *°*C cooler than built-up categories on a hot day. Close to 75% of dense forest, 75% of water, 60% of low plants and 40% of sand LCZ pixels recorded moderate heat stress on a very hot day in the hot season. On the other hand, about 35% of lightweight low-rise LCZs were below the lower limit of the strong heat stress category. In general, about 75% of built-up LCZs recorded strong to very strong heat stress on a very hot day in the hot season. Of this, about 20% of compact high-rise, lightweight low-rise and large low-rise areas recorded very strong heat stress on the same day. The sparsely built-up open low-rise LCZ was not entirely an exception, as about 10% of pixels in this category recorded very strong heat stress.

### 3.9 Local Climate Zones in Very Strong Heat Stress Areas

Very strong heat stress was recorded on a hot day in 3255 ha of the municipality (Table 7) with most of the sites in the eastern areas where residential and commercial built-up areas are located. The greatest proportion of these sites were in the densely built-up lightweight low-rise (LCZ 7) followed by compact high-rise areas (LCZ 1). Compact high-rise and large low-rise occupy less than 2% of the study area but contained the largest proportion (51.3%) of the strongest heat stress locations. About 73% of the city is occupied by open low-rise and low plants with sparse or no buildings, which constituted close to 36% of the strongest heat stress areas. Soil/sand and water, which are either covered by water, have a high water table or are close to a water body, had the lowest proportion of the strongest heat stress areas (3%).

## 4 Discussion

This study has shown that local climate Zones can be mapped onto the rural–peri urban–urban continuum with high accuracy by applying the WUDAPT procedure on multi-date and multi-band Landsat 8- and Landsat 9-reflective data. The high quality of input remotely sensed data from Landsat 8 and Landsat 9, and representative training data, as well as the proven high performance of the random forest classifier [37,56,57] resulted in high mapping accuracy. The Landsat 8 and Landsat 9 images used in this study have high spectral resolution, a high signal-to-noise ratio, and high radiometric resolution [58–60], which aid image classification. The use of multi-temporal imageries incorporates seasonal variations as additional information which combines with spectral resolution from the wavelength ranges used to enhance the discriminability of classes. Previous studies using Landsat and other remotely sensed datasets have also shown that using multi-temporal data enhances classification accuracy when compared to the application of a single-date imagery [61–66]. Additionally, the random forest classifier is known to outperform other approaches when multi-dimension data are used [67]. The high quality of training data can be supported by TDSI values exceeding 1.5; as values close to 2 imply that the two classes compared can be separated with ease. This study confirmed that existing land cover maps can be combined with high-resolution Google Earth data to generate training samples.

The sea influences development patterns as well as the configuration of LCZs, concentrating economic activities and built environments along the coast, especially near the harbor within the study area. There is less development to the west compared to the east of the municipality. This resulted in spatial variability in the LCZ arrangement, with resulting spatial heterogeneity in LSTs and thermal comfort/stress. Lightweight low-rise occupied a lower proportion of the municipality than open low-rise areas. Lightweight low-rise areas have densely packed buildings with a low floor area and high population density, exposing communities with low adaptive capacities to heat health risks [68,69]. Low-income communities congest in these areas as they lack resources to occupy spacious settlements in open low-rise areas, due to the high cost per unit area of land [69]. The bare soil LCZ occupied the smallest proportion of all the categories in the municipality. The beach areas could also be reserved for tourism and leisure purposes as they typically have a higher socioeconomic value to the municipality. However, these areas have few buildings in place such as shops, food outlets and offices that support tourism, leisure and other businesses. The low plants LCZ occupied the largest proportion in the municipality. Sutherland et al. [70] also indicated that the municipality hosts a continuum from deep rural to high-density urban with sparsely built-up rural dominating the northwest and southwest. Open grasslands within the built-up LCZs are large enough in coverage to be separated from the surrounding categories.

The thermal data of Landsat 8 can be adjusted to map land surface temperature and thermal comfort variability at times other than the overpass. In eThekwini municipality, the overpass was in the morning while the study was interested in thermal comfort at peak temperature times of the day in order to assess risks. The adjusted temperatures, based on a local weather station, allowed us to map daytime thermal comfort at the peak temperature time on very hot days in the cool and hot season, which would have been supposedly impossible if directly using Landsat 8 and Landsat 9 thermal data. This is significant for spatially explicit regional-scale monitoring of heat stress using available satellite datasets.

Due to the low insolation levels in the cool dry season, both the built-up and vegetation based LCZ have low temperatures with low interclass variations. In the hot season, the evaporative cooling effect of vegetation will be pronounced and vary with vegetation type [25]. This pattern of warmer LST in the hot than in the cool seasons is common and has been attributed to temporal variations in solar radiation received [23,36,71,72]. Simultaneously, LCZs with high heat absorption capacities will be exposed to high insolation levels resulting in high surface temperatures and large interclass variations, especially when compared with non-built-up LCZs. Land surface temperature was found to be higher in the built-up than other LCZs, especially in the hot season. Land surface temperatures increased with the height and density of buildings as well as the imperviousness of the surroundings. As a result, compact high-rise areas were the warmest followed by large low-rise areas, in all seasons. The highest land surface temperatures in compact high-rise areas were attributed to high heat absorption by the buildings, a low sky view factor and the impediment of the heat removal effect of winds by buildings due to friction, which lowers wind speed and strength [28,73]. Like in large low-rise areas, the proportion of impervious surfaces is also very high in compact high-rise areas.

The study also made it possible to assign heat stress to LCZs which enables monitoring and measurement of heat risk that is inherent to different LCZs. Risk can be related to daytime temperatures and be used to develop early warning systems to alert people about heat stress risk in different LCZs in hot seasons. In another study [74], the entire eThekwini Municipality was projected into the extreme caution category (32 to 38 *°*C) under future climate change, while the current analysis showed variations between and within LCZs across the area with some locations already experiencing very strong heat stress. Capturing the fine scale heterogeneity in heat stress is significant for place-specific intervention measures. A daytime thermal comfort map was produced which indicated that the conditions remained largely comfortable for the entire municipality during the daytime in the cool season. On the other hand, the entire municipality recorded moderate to very strong heat stress during a hot afternoon in the hot season, especially in densely built-up LCZs. In the city of Tel Aviv, Israel, Mandelmilch et al. [73] also noted that insolation levels influenced heat exposure, as variation was remarkable during the hot hours of the day. This implies intolerable thermal conditions for outdoor activities in strong to very strong heat stress areas requiring alerts and mitigation. Moderate heat stress levels observed in the southern and northern areas, which correspond with non-built LCZs (low plants, dense trees and sparsely built [rural]), highlights their value in heat mitigation and as thermal refuges. This indicated that on a very hot day, even non-built-up LCZs experience thermal stress but to a lesser extent than the built-up LCZs. Thermal discomfort increased from west to east, generally indicating the influence of surface alteration associated with replacing natural cover with buildings and other artificial high-heat absorption materials. Wu et al. [75] also observed that rural temperatures were elevated in intense heat cases. The findings concur Lau [76] who note that variations in the morphology of urban areas do not only affect micro-climates, but also the occupants’ thermal sensation.

Heat stress can be mapped along the rural–peri-urban–urban gradient allowing an understanding of differential necessary responses as well as of the importance of rural and peri-urban areas for heat reduction in cities. The rural–urban temperature difference was stronger on an extremely hot day in the hot season than on an extremely cool day. The small rural–urban LST difference in the cool season could be a consequence of low plant and soil water levels during the dry season, which reduce the cooling effect of surfaces. Additionally, some low plants dry out in the dry season. The thermal conductivity of dry surfaces was also found to compare to that of urbanized areas in Ouagadougou in Burkina Faso [77]. Scant vegetation cover was also found to resemble similar thermal characteristic as built-up areas in Kuwait [78]. The findings also concur with Lee et al. [79] who observed higher temperatures in urban rather than rural areas in Xuzhou. According to McCarthy et al. [80], high anthropogenic heat levels in urban areas also contribute to the elevated temperatures compared to rural areas. Similarly, in eThekwini Municipality, industrial activities and emissions, such as from vehicles, dominate the eastern urbanized area, which may also contribute to the temperature difference with rural areas. The variations in rural–urban temperature with season was also observed in Beijing, where temperatures were high in spring, summer and autumn, and low in winter [2]. The analysis for the hot season in this study was performed on an extremely hot day, which elevates differences as rural and urban areas absorb heat at different rates. Wu et al. [75] also observed that rural–urban temperature differences were elevated in cases of intense heating in Taipei in Taiwan and Yilan in Japan. In this study, rural–urban temperature differences could reach approximately 6 *°*C, while the difference even reached 10 *°*C in Rotterdam in the Netherlands. The differences in temperature indicate that rural areas offer a natural ecosystem service which has strong heat mitigation value under extremely high temperatures [81,82].

The findings of this study showed that thermal stress responds to development trajectories. The choice of the spatial structures of built-up and landcover based LCZ types has a strong bearing on the extent of thermal stress experienced by citizens as well as benefits attained from low heat absorption LCZs. While climate change policies and strategies in most cases have emphasized water resources and agriculture, a focus on thermal variability and stress has proved to be equally important. At the same time, strategies need to be varied and customized to area-specific thermal behaviors as a uniform approach will disadvantage locations that are most at risk. For example, in eThekwini Municipality, heat mitigation activities proved to be more necessary in densely built-up and populous areas. The findings could guide provincial and municipal authorities to locate areas where assessments of adaptation and mitigation activities should be carried out. The findings also indicated that authorities need to mobilize resources to enable communities in high heat vulnerability areas to cope with extremes. Additionally, the method used in this study expressed the need to increase the number of high-temporal-resolution thermal comfort monitoring instruments to improve the accuracy of observations and models in eThekwini municipality. Furthermore, policies and strategies for conserving vegetation and water ecosystems need to be strengthened as they have proved to be of high heat mitigation value in the municipality. This should include promotion of greening efforts including roof-top vegetation.

## 5 Conclusions

Based on these findings, the study drew the following conclusions:

- Multi-spectral and multi-temporal Landsat 8 and Landsat 9 data effectively and accurately map LCZs and their link with land surface temperature across the rural– urban gradient;
- The land surface temperatures can be adjusted through data integration techniques to overcome the limitation of temporal resolution and overpass time. This enables monitoring and assessment of the link between LCZs and LST at times of peak temperatures;
- Adjusted LSTs enable the monitoring and understanding of heat stress variations between LCZs and along the rural–urban gradient;
- Using spatial overlays, heat stress can be assigned LCZs to quantify their role towards heat elevation or heat mitigation. Based on this, it was concluded that rural areas, vegetation areas (forests and low plants) and wetlands are important for heat reduction and thermal comfort moderation along the rural–urban gradient;
- Heat stress extremes should be closely monitored in densely built-up LCZs for possible issuance of alerts and mitigation efforts on very hot days. Heat stress can also be experienced in some areas within densely built-up LCZs even on a hot day during the cold season;
- An early warning system can be developed based on approaches used in this study to alert people about heat stress in different LCZs.

Overall, the study enhances the understanding of differential responses to heat stress in LCZs under temperature extremes along the rural–urban gradient. The study recommends validating the physical comfort conditions with the lived experiences of the communities in the area. Community-based analysis approaches which include understanding local people’s perceptions and experiences of the thermal environment will assist in validating physical approaches. The findings of this study present the spatial patterns in eThekwini, implying the need for similar studies in other areas as findings cannot be generalized to the entire country. Future studies can benefit from the installation of high-resolution thermal comfort measuring instruments which are fairly distributed to capture experiences in different parts of the country as well as to improve remotely sensed data. Urban greening policies and strategies need to be upscaled and promoted in the municipality due to the high heat reduction value of vegetation-based LCZs. Studies can also investigate the implications of changes in LCZs on future thermal comfort.

## Figures and Tables

**Table 1:** Landsat data used for local climate zones and land surface temperature mapping in eThekwini Municipality.

Landsat Mission	Path/Row	Date Acquired	Season
Landsat 8	168/80 and 168/81	24 January 2022	Hot wet
Landsat 8	168/80 and 168/81	9 February 2022	Hot wet
Landsat 8	168/80 and 168/81	23 March 2022	Hot wet
Landsat 8	168/80 and 168/81	29 March 2022	Hot wet
Landsat 9	168/80 and 168/81	24 May 2022	Cool dry
Landsat 9	168/80 and 168/81	9 June 2022	Cool dry
Landsat 8	168/80 and 168/81	19 July 2022	Cool dry
Landsat 8	168/80 and 168/81	20 August 2022	Cool dry

**Table 2:** Representative pictures of samples extracted from GoogleEarth within the eThekwini Municipality.

Local Climate Zone	Local Climate Zone
Baresoil/Sand	Open low-rise
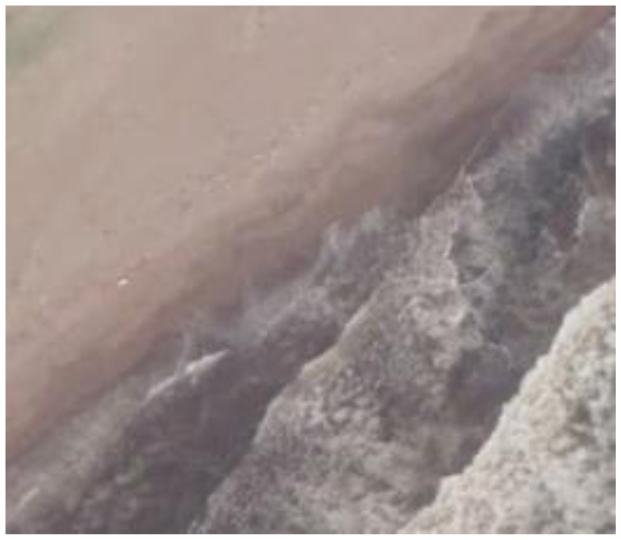	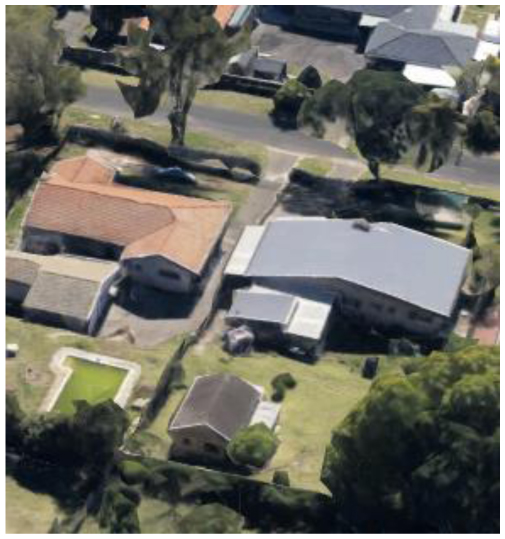
Low plants	Lightweight low-rise 1
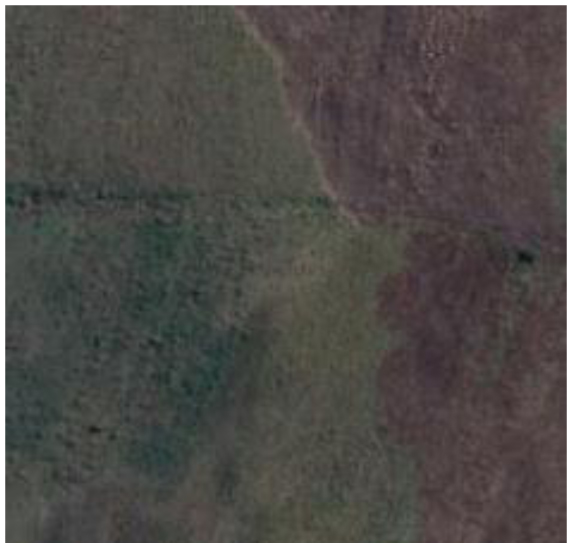	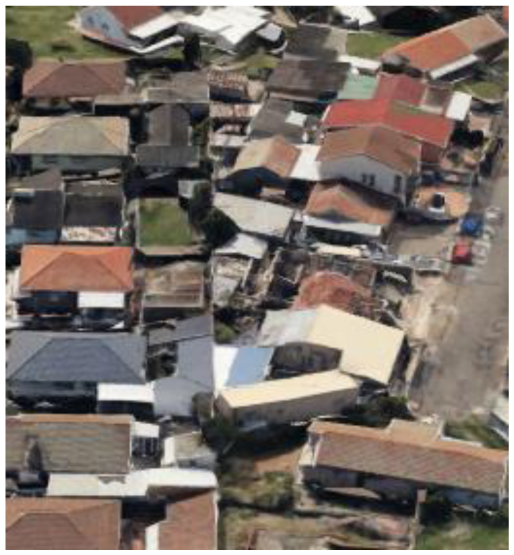
Water	Light weight low-rise 2
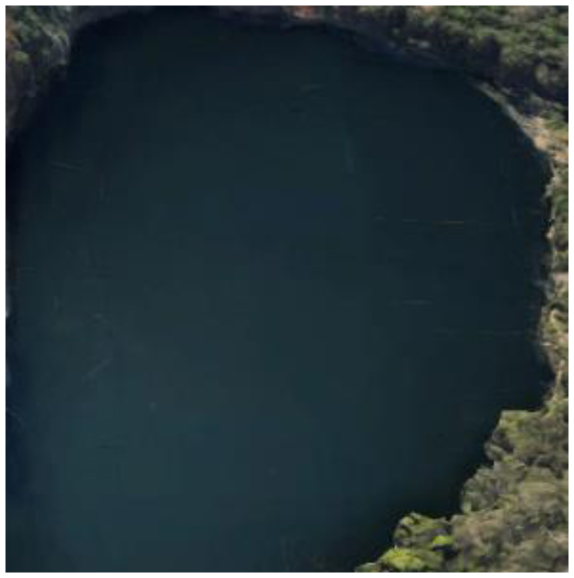	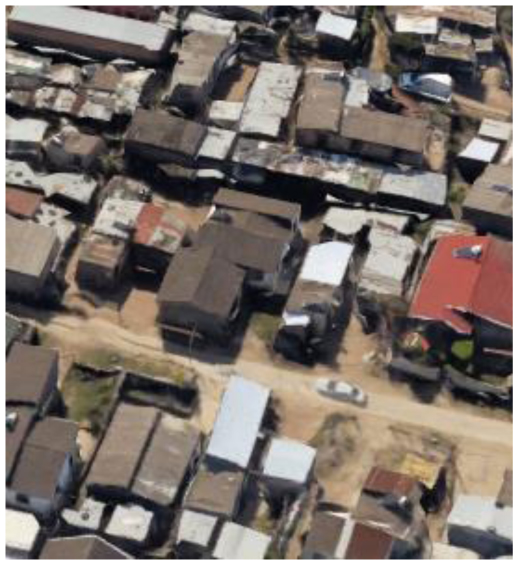
Dense forest	Large low-rise
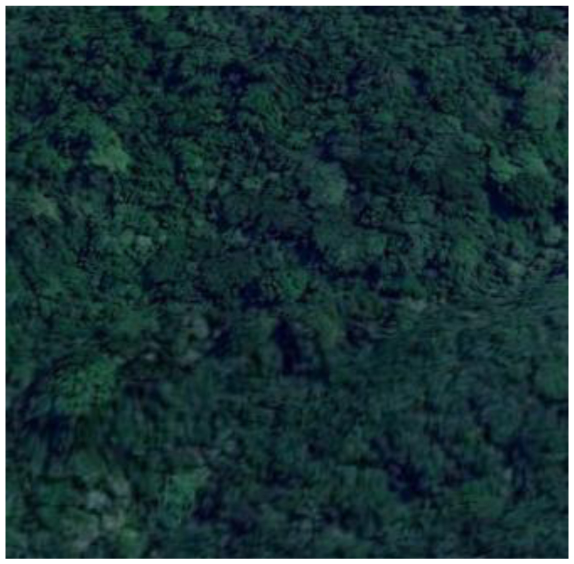	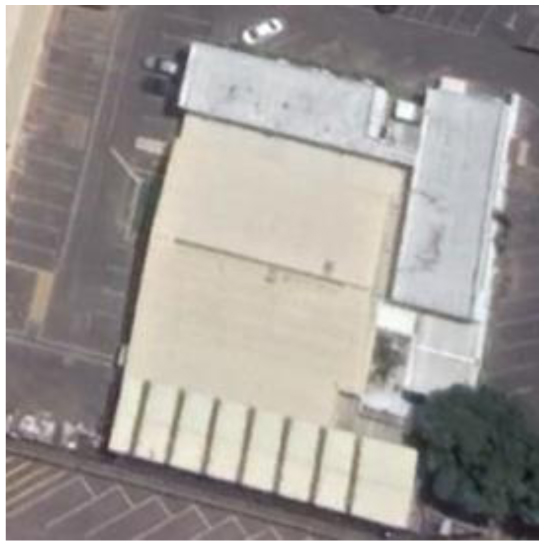
Sparsely built-up	Compact high-rise
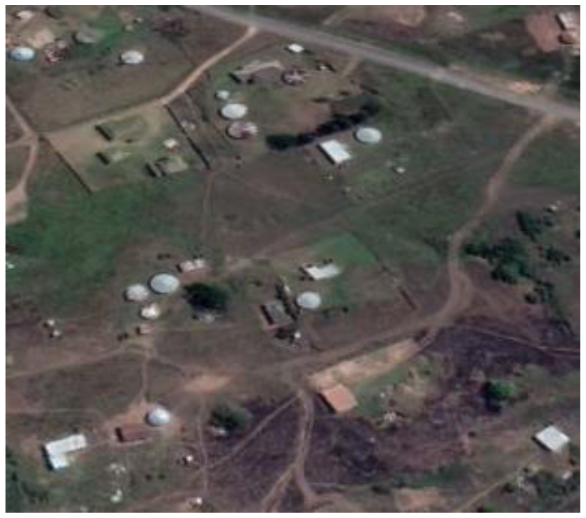	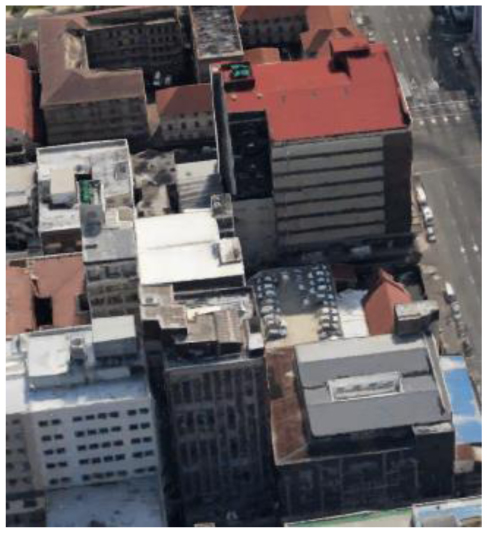

**Table 3:** Proportions of eThekwini Municipality occupied by LCZ categories.

Local Climate Zone	Area (Hectares)	Proportion of Municipal Area (%)
Compact high-rise (LCZ 1)	4650	1.82
Open low-rise (LCZ 6)	36,032	14.09
Lightweight low-rise (LCZ 7)	16,941	6.63
Large low-rise (LCZ 8)	1322	0.52
Dense forest (LCZ A)	44,822	17.53
Low plants (LCZ D)	146,529	57.32
Bare soil/Sand (LCZ F)	990	0.39
Water (LCZ G)	4365	1.71

**Table 4:** Accuracy of local climate zone mapping using the random forest classifier on Landsat 8 and Landsat 9 data.

Local Climate Zone	Producer Accuracy (%)	User Accuracy (%)
Bare soil/sand	91.2	96.1
Dense forest	82.9	92.6
Lightweight low-rise	96.7	83.3
Low plants	92.3	84.6
Large low-rise	64.9	93.1
Compact high-rise	74.5	88.8
Open low-rise	74.9	88.5
Water	98.8	99.4

**Table 5:** Contribution of inter-class separability to mapping accuracy.

	LCZ1	LCZ6	LCZ7	LCZ8	LCZA	LCZD	LCZF	LCZG
LCZ1	-	1.94	1.60	2.00	2.00	1.95	2.00	2.00
LCZ6		-	1.57	1.72	2.00	1.69	2.00	2.00
LCZ7			-	1.96	2.00	1.93	2.00	2.00
LCZ8				-	2.00	1.90	1.95	2.00
LCZA					-	2.00	2.00	2.00
LCZD						-	2.00	2.00
LCZF							-	2.00
LCZG								-

**Table 6:** Adjustment of average LSTs across the image to correspond with daily peak temperature as measured at Ushaka.

Date	Temperature at 0950 Hours (*T _0950 hours_*)	Maximum Temperature (T_max_) (°C)	T_max_ Minus *T _0950 hours_* (°C)
24 January	27.7	29.5	1.8
**9 February**	**27.2**	**31.9**	**4.7**
23 March	25.4	27.8	2.4
29 March	25.6	28.6	3.0
24 May	21.6	24.4	2.8
9 June	19.1	25.0	5.9
19 July	20.2	23.0	2.8
**20 August**	**19.3**	**22.6**	**3.3**

In bold are the coldest and warmest days that were used for further analysis of thermal comfort.

**Table 7:** Extent of strongest heat stress areas in eThekwini Municipality.

LCZ	Very Strong Heat Stress Area (ha)	Proportion of Total Very Strong Heat Stress Area (%)	LCZ Coverage of Entire Study Area (%)
Compact high-rise	694	21.3	1.82
Open low-rise	496	15.2	14.09
Lightweight low-rise	220	6.8	6.63
Large low-rise	977	30.0	0.52
Dense forest	96	2.9	17.53
Low plants	675	20.7	57.32
Soil/sand	20	0.6	0.39
Water	79	2.4	1.71

## Data Availability

All data available upon request.
